# The Effect of Combining Transcranial Direct Current Stimulation and Pain Neuroscience Education in Patients With Chronic Low Back Pain and High Pain Catastrophizing: An Exploratory Clinical, Cognitive, and fMRI Study

**DOI:** 10.1002/brb3.70543

**Published:** 2025-05-08

**Authors:** Cory Alcon, Sarah Margerison, Haley Kirse, Christopher Zoch, Paul Laurienti, David Seminowicz, Sharon Wang‐Price

**Affiliations:** ^1^ Department of Physical Therapy High Point University High Point North Carolina USA; ^2^ Department of Neural and Pain Sciences University of Maryland School of Dentistry Baltimore Maryland USA; ^3^ Center to Advance Chronic Pain Research University of Maryland Baltimore Baltimore Maryland USA; ^4^ Laboratory for Complex Brain Networks, Wake Forest School of Medicine Winston‐Salem North Carolina USA; ^5^ Department of Medical Biophysics Schulich School of Medicine & Dentistry University of Western Ontario London Ontario Canada; ^6^ School of Physical Therapy Texas Woman's University–Dallas Dallas Texas USA

**Keywords:** cognition, fMRI, low back pain, pain catastrophizing, tDCS

## Abstract

**Objectives:**

Priming the neural circuitry likely targeted by pain neuroscience education (PNE) using transcranial direct current stimulation (tDCS) may enhance the efficacy of PNE. This exploratory study aimed to examine the effects of combining tDCS and PNE in those with chronic low back pain (CLBP).

**Methods:**

Six participants experiencing CLBP completed the study. Assessments for pain catastrophizing, kinesiophobia, pressure pain thresholds (PPT), pain intensity, cognitive function, and resting state and task fMRI were collected before and after the combined tDCS and PNE intervention. Each participant received five 20‐min sessions of 2.0 mA tDCS targeting the left dorsolateral prefrontal cortex (DLPFC), followed by a 20‐min PNE session.

**Results:**

The participants had a 58.9% reduction in pain catastrophizing, a 25.9% reduction in kinesiophobia, and an 18.8% improvement in cognitive function (i.e., reduced attentional interference). The MRI results indicated increased gray matter volume within the left DLPFC. Additionally, larger reductions in DLPFC activity at rest were associated with larger reductions in kinesiophobia. Increased modularity within networks responsible for cognitive control and executive functions was evident following the intervention.

**Discussion:**

Our clinical and fMRI outcomes shed light on the clinical potential of combining tDCS and PNE, as well as the mechanisms substantiating their effects. We speculate that tDCS alters brain activity and structure, amplifies the effects of PNE, and promotes positive changes in the cognitive‐evaluative and sensory‐discriminative pain domains investigated. A randomized controlled trial is warranted to determine the effects of tDCS combined with PNE compared with tDCS or PNE alone.

## Graphical abstract

Participants with chronic low back pain and high pain catastrophizing underwent five sessions of tDCS to the left DLPFC and Pain Neuroscience Education (PNE). Participants showed increases in gray matter density and functional connectivity as well as improvements in pain behaviors and cognitive performance. Our clinical and fMRI outcomes shed light on the clinical potential of combining tDCS and PNE, as well as the mechanisms substantiating their effects.

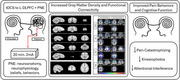

## 1 Introduction

Maladaptive cognitive and affective behaviors such as pain catastrophizing and kinesiophobia are commonly associated with the presence of persistent pain (Huysmans et al. 2018; Marshall et al. 2017). When these behaviors go unnoticed or unaddressed, the likelihood of poor outcomes rises dramatically, including increased opioid misuse (Lazaridou et al. 2017), greater physical disability (Picavet et al. 2002), heightened prevalence of anxiety/depression (Edwards et al. 2011), and increased healthcare expenditures (de Boer et al. 2012). Pain catastrophizing and kinesiophobia have been found to predict the development of chronic pain and are hypothesized to be the underlying cause of recalcitrant responses to management strategies in patients with chronic pain (Darnall 2016; Farin 2015; Oosterhaven et al. 2019; Zhaoyang et al. 2020).

In recent years, research has been directed to interventions that aim at centrally mediated factors (Balagué et al. 2012). One of these strategies, pain neuroscience education (PNE), is a cognitive‐based intervention used to help patients reconceptualize their understanding of the pain experience and positively alter negative beliefs and pain cognitions associated with pain catastrophizing and kinesiophobia (Amer‐Cuenca et al. 2020). Systematic reviews have demonstrated the efficacy of PNE in addressing pain catastrophizing, kinesiophobia, avoidance behaviors, and healthcare utilization for patients with chronic pain (Louw et al. 2016). However, evidence also demonstrates that patients become less responsive to PNE when they reach higher levels of pain catastrophizing and kinesiophobia (Birch et al. 2020; Malfliet, Van Oosterwijck, et al. 2017; Pagé et al. 2017). As patients with chronic pain display dysfunction in cognitive domains such as attention and memory, limited cognitive reserve may reduce one's ability to evaluate, interpret, and revise maladaptive behaviors (Kreitler and Niv 2007; Melkumova et al. 2011; Moriarty et al. 2011). This may make interventions like PNE less effective in those with high versus low pain catastrophizing. Therefore, PNE alone is likely insufficient to treat chronic pain patients with high catastrophizing.

Considering the central barriers that may limit the success of PNE, efforts to modulate these factors are needed. Transcranial direct current stimulation (tDCS) is a noninvasive brain stimulation technique used to alter the excitability of the brain regions being targeted. As such, tDCS could have the potential to make subsequent cognitive‐based therapy, such as PNE, more effective. A number of studies have examined the use of tDCS in isolation on pain, particularly targeting the primary motor cortex (M1) (Ihle et al. 2014; Lefaucheur et al. 2006; Morya et al. 2019). Although several studies have shown a positive effect of tDCS targeting the M1 on pain in those with fibromyalgia, migraine, and spinal cord injury, there appear to be minimal effects in those with CLBP (Baptista et al. 2019; Hazime et al. 2017; Mehta et al. 2015; Pinto et al. 2018; Schabrun et al. 2018; Shirahige et al. 2016; Wen‐Hsuan Hou 2016; Zhu et al. 2017). Research supports the use of tDCS for improving attention, memory, and learning in cognitively impaired and pain‐suffering participants when the dorsolateral prefrontal cortex (DLPFC) is targeted (Brasil‐Neto and Fregni 2012; Silva et al. 2017; Soltaninejad et al. 2019). In addition, tDCS has been shown to reduce pain and improve pain catastrophizing and executive function/attention deficits in patients with chronic pain (DalĺAgnol et al. 2015; Pinto et al. 2018; Silva et al. 2017). Therefore, if attentional resources are low, increasing the cortical excitability via tDCS could support improved consumption of PNE for greater behavioral effects that lead to longer‐lasting and more impactful cognitive change (Perrotta and Perri 2022; Schabrun and Chipchase 2012; Seminowicz and Čeko 2015). However, the effects of combining these two interventions on maladaptive pain behaviors and cognitive impairments simultaneously have not yet been investigated.

Therefore, the primary objective of this exploratory study was to examine pain‐related behaviors and cognitive function in participants with CLBP and high pain catastrophizing after receiving combined tDCS and PNE. The secondary purpose of this study was to describe structural and functional brain changes following the intervention and their associations with primary outcome changes. Finally, the relationships between cortical activity and pain behaviors as well as cortical activity and cognitive function were also examined.

## 2 Materials and Methods

### 2.1 Design

This study was conducted between November 2022 and March 2023 at High Point University (HPU) and Wake Forest University (WFU) in North Carolina (NC), United States, and Texas Woman's University—Dallas (TWU), Dallas, Texas (TX). Ethical approval was obtained by the Institutional Review Boards at HPU, WFU, and TWU. The trial was also registered with ClinicalTrials.gov (NCT05571215).

### 2.2 Participants

Six participants, age 36.7 ± 12.9, with reports of CLBP and Pain Catastrophizing Scale (PCS) scores ≥ 30 were enrolled in this explorative study. CLBP was defined as a back pain problem (between the 12th rib and sacroiliac joint) that has persisted for at least 3 months and has resulted in pain on at least half the days in the past 6 months (Bernstein et al. 2012). A score of ≥ 30 on the PCS is an accepted threshold indicating clinically relevant levels of pain catastrophizing (Osman et al. 1997; Osman et al. 2000; Sullivan et al. 1995). Exclusion criteria included a history of previous low back surgery, systemic joint disease (e.g., rheumatoid arthritis), evidence of red flags (e.g., fracture, infection, tumor, cauda equina syndrome), cancer, neurological disorders, neuropathy, Raynaud's disease or pregnancy, an inability to maintain the testing and treatment positions (i.e., sitting for > 30 min), or an inability to undergo MRI. Additional exclusion criteria for the tDCS included (1) a history of significant head trauma, (2) electrical, magnetic, or mechanical implantation (e.g., cardiac pacemakers or intracerebral vascular clips), (3) a metal implant in the head or neck areas, (4) a history of seizures or unexplained loss of consciousness, (5) an immediate family member with epilepsy, (6) the use of seizure threshold lowering medicine, (7) the current abuse of alcohol or drugs, and (8) a history of psychiatric illness requiring medication controls. Participants were recruited on campus at HPU and in local clinics in the NC Triad region. An a priori power analysis was not performed because this is an exploratory study.

### 2.3 Outcome Measures

All outcomes were collected before and after completion of the intervention protocol from each participant. The outcome measures consisted of three domains: pain, cognitive function, and structural/functional brain measures.

#### 2.3.1 Pain Outcomes

Pain outcomes included the PCS, Tampa Scale of Kinesiophobia (TSK), Central Sensitization Inventory (CSI), Numeric Pain Rating Scale (NPRS), and Pressure Pain Thresholds (PPTs). The PCS (scores ranging from 0 to 52) was used to qualify the participants’ levels of pain catastrophizing and also to measure change in catastrophizing after completion of the study (Sullivan et al. 1995). The TSK (scores ranging from 17 to 68) was used to measure levels of movement‐related fear (Vlaeyen et al. 1995). The CSI (scores ranging from 0 to 100) was used to quantify the overall sensitivity of the nervous system via a broad base of questions regarding both nociceptive and emotional responses to stimuli (Mayer et al. 2012). This allowed for a more comprehensive, multifactorial understanding of the pain process. The NPRS was used to assess the participant's current pain intensity from 0 (*no pain*) to 10 (*worst imaginable pain*) (Childs et al. 2005). Finally, PPTs were performed to determine the minimum pressure that induced pain (Zicarelli et al. 2021). PPTs provide another measure of pain sensitivity and nociceptive function and allow for a direct reflection of changes in tolerance to pressure stimuli (Farasyn and Meeusen 2003). With the participant in the prone position, PPT was performed by pressing a pressure algometer (Wagner Instruments, Greenwich, CT) at a steady state into the most tender point of the participant's lower back. The participant was asked to signal to stop the testing when the applied pressure became painful (i.e., pain threshold). Three trials were performed, and the average of the second and third trials in newtons (N) was used for data analysis. To limit the effects that PPT testing may have had on self‐reported questionnaires and cognitive testing, PPTs were performed last.

#### 2.3.2 Cognitive Outcomes

Cognitive outcomes included the computerized Stroop color‐word test (SCWT) and the comprehensive trail‐making test—second edition (CTMT2). The SCWT consisted of three trials (i.e., Stroop 1−3) that measured attentional interference by collecting participants’ reaction times to 24 visual stimuli in each trial (Golden 1978; Scarpina and Tagini 2017). In Stroop 1, participants stated the word displayed on the computer screen in black ink (red, yellow, blue, or green). In Stroop 2, participants stated the color of the square presented on the screen (red, yellow, blue, or green). Finally, in Stroop 3, participants were presented with the name of a color, written in a different font color. They were asked to state the color of the font rather than the word presented. This study utilized the computerized version via the software program DirectRT (Empirisoft Corp., New York, NY). Participants responded to stimuli verbally through a headset that captured individual reaction times for each stimulus. The CTMT2 involved a series of trials requiring cognitive control between competing stimuli and schemas to complete respective trials (Reynolds 2020). Trials 1−3 measured inhibitory control by completing trials with increasing numbers of distractors. Trials 4−5 measured set‐shifting by requiring participants to shift between opposing mental sets (i.e., numbers and letters). For each of the cognitive tests, participants were provided standardized instructions.

#### 2.3.3 MRI Acquisition

MRI scans were performed within 1 week prior to initiation of the intervention protocol and within 48 h of completion of the intervention protocol. MRI imaging data was collected at Wake Forest School of Medicine using a single 3T Siemens Skyra scanner equipped with a 32‐channel head coil, response button box, and a rear projection screen. While in the scanner, participants completed a structural MRI, resting‐state fMRI, and Flanker task‐based fMRI. T1‐weighted structural imaging data were acquired using a 3D MPRAGE GRAPPA2 sequence (Echo time [TE] = 2.98 ms, repetition time [TR] = 2.3 s, 192 slices). Following structural acquisition, resting‐state blood‐oxygenation level‐dependent (BOLD) and functional imaging data were sequentially acquired in the transverse plane using an echo‐planar imaging sequence (TE = 25 ms, TR = 2.0 s, spatial resolution = 4.0 mm × 4.0 mm × 5.0 mm, slice thickness = 5.0 mm, flip angle = 75°, 35 slices per volume, acquisition time = 6 min and 20 s). For the task‐based scan, the Flanker task was administered using E‐Prime 3.0 with the same imaging acquisition protocol as the resting‐state scan. The MRI scanner is equipped with a rear projection screen and head coil affixed mirror that was used to present the fixation point during the resting state and the Flanker task.

### 2.4 Intervention

Each participant received five sessions of tDCS and NPE over 2 weeks. The first intervention session was administered immediately following the completion of the baseline testing, and post‐intervention assessments were performed after the fifth or last intervention session. The location of each participant's left DLPFC was determined using the Beam F3 measurement system with the participant in sitting (Beam et al. 2009). Two 5 cm × 5 cm sponge electrodes soaked in sterile saline were applied to the left DLPFC and to the right supraorbital region. Each participant received 2 milliampere (mA) of anodal tDCS targeting the left DLPFC for 20 min continuously plus a 30‐s ramp‐up at the beginning of the stimulation and a 30‐s ramp‐down following the stimulation (DC Stimulator, Neuroconn GmBh, Ilmenau, Germany). The parameters used were consistent with the current literature on tDCS for pain (Lefaucheur et al. 2017).

Immediately following the completion of each tDCS intervention, participants were given a one‐on‐one PNE session provided by the researcher (CA), who had specific expertise in the content and utilization of this technique in the clinical setting. Each educational session lasted approximately 30 min (Galán‐Martín et al. 2019). An outline of the topics covered over the course of the study is found in Table 1. Throughout each session, participants were asked questions regarding their understanding of the session topic in order to individualize the PNE within the confines of the guided topics. Each session began with a brief review of the concepts discussed in the previous session and time for the participant to ask clarifying questions.

**TABLE 1:** Outline of pain neuroscience education sessions.

Session no.	Basic contents
Session 1	Pain as an alarm system. Pain is not equal to damage. Psychosocial and cultural aspects of pain.
Session 2	Differences between acute and chronic pain Danger assessment system. Amplification and inhibition systems. Pain as a brain response.
Session 3	Consequences of chronic pain and central sensitisation. Movement, motor control disorder, and kinesiophobia. Fear‐avoidance behaviours. Pain catastrophizing. Structural and functional disturbances that generate persistent pain.
Session 4	Knowledge as a tool in the evaluative process of sensory stimuli. The belief system and its epigenetic effects. Reversibility of structural and functional changes. Neuroplasticity mediated by cognitive and somatosensory stimuli, and physical exercise.
Session 5	Review of the contents covered in the first four sessions, and of the most relevant aspects of the PE group sessions.

### 2.5 Data Analysis

#### 2.5.1 Pain Outcomes

All pain outcomes, including the PCS, TSK, CSI, and NPRS, were completed by the participants and scored by a member of the research team based on the respective outcome scale. PPT values were documented for each of the three trials in N, and the last two measurements were averaged and used for data analysis (Farasyn and Meeusen 2003). Wilcoxon signed‐ranked tests were performed to assess whether the changes in pain and pain behavior outcomes were statistically significant following the intervention protocol, with *p* < 0.05.

#### 2.5.2 Cognitive Outcomes

Each SCWT trial was processed to obtain a reaction time for all participants. Audio recordings were reviewed to determine the accuracy of each stimulus on each trial. Individual stimuli with incorrect responses were not included when determining the mean reaction times (ms) and standard deviations (SD) for each trial. Minimum and maximum bounds were set at ±3 SDs from the mean. Any data point outside of the three SDs from the mean was denoted as an outlier and removed, and then a revised mean and SD were calculated. Finally, the ms from Stroop 1−3 were used to calculate an interference score using the following equation: interference score = Stroop 3 − ([Stroop 1 + Stroop 2]/2) (Valentijn et al. 2005; Van Der Elst et al. 2006). Thus, each participant had one interference score before and after the completion of the intervention protocol.

For the CTMT2, the time required to complete each trail of the CTMT2 was recorded for each participant in seconds (s). The times for each trial were used to identify a *t*‐score that corresponded to the participant's current age based on normative data (Reynolds 2020). The *t*‐scores from Trials 1−3 and 4−5 were summed separately, and *t*‐scores were determined for those sums as an inhibitory control index and a set‐shifting index, respectively. Therefore, two scores culminated from the completion of the CTMT2. Three separate Wilcoxon signed‐ranked tests were performed to assess whether the changes in cognitive outcomes were statistically significant following the intervention protocol, with *p* < 0.05.

### 2.6 Voxel‐Based Morphometry Preprocessing and Analysis

Structural data was preprocessed using the Computational Anatomy Toolbox (CAT12) for Statistical Parametric Mapping (SPM12) (http://www.neuro.uni‐jena.de/cat/). Segmentation, normalization, and interpolation to a 1.5 mm voxel size were performed on T1 images of all participants for both the pretreatment and posttreatment scanning sessions. Data was smoothed with an 8 mm full width at half maximum (FWHM) Gaussian kernel.

Voxel‐based morphometry (VBM) uses segmented high‐resolution structural data to determine the amount of gray or white matter in each voxel and is often used to compare the amount of gray or white matter between groups (Ashburner and Friston 2005, Ashburner and Friston 1999). The CAT12 toolbox in SPM12 was used to perform VBM analysis comparing gray matter within structural scans of each participant before and after treatment, using intracranial volume as a covariate to control for differences in brain size among the participants.

An initial whole‐brain analysis was performed using a clusterwise significance threshold of *p* < 0.05 with family‐wise error (FEW) correction. This analysis was followed by a targeted approach using the DLPFC as an apriori region of interest (ROI) using an uncorrected threshold of *p* < 0.001. ROIs are used to constrain fMRI analysis to contiguous groups of voxels representing a functional region of the brain. A 10 mm sphere centered at −45, 33, 24 was generated via MarsBaR to represent the left DLPFC (Brett et al. 2002), reflecting the peak voxel of a DLPFC region previously identified as showing increased cortical thickness following successful treatment of CLBP (Seminowicz et al. 2013).

The DLPFC ROI was used because it represents the target of the tDCS treatment. Additionally, previous studies have shown that the DLPFC of people with CLBP increased in thickness following successful treatment (Seminowicz et al. 2013). and that cognitive behavioral therapy reduced pain catastrophizing and increased DLPFC thickness in people with general chronic pain (Seminowicz et al. 2013).

### 2.7 fMRI Processing and ALFF Calculation

The amplitude of low‐frequency fluctuation (ALFF) refers to the power of the BOLD signal after band pass filtering from 0.01 to 0.08 Hz and can be used as a proxy for neural activity within a brain region at rest (Zuo et al. 2010). After filtering, the root mean square at each voxel is taken (Yang et al. 2007).

First, resting state data was preprocessed using SPM12 (Wellcome Trust Centre for Neuroimaging, London, UK). This included slice timing correction, realignment, coregistration, normalization to a standard template, and interpolation to a 2 mm × 2 mm × 2 mm voxel size using a fourth‐degree B‐spline. Data was smoothed with an 8 mm FWHM Gaussian kernel. Motion was assessed using a custom program. Average framewise displacement was determined for each scan (mean: 0.33 mm, range: 0.12–0.55). We did not exclude participants for excessive motion due to the limited number of participants in the scan group; however, framewise displacements were compared between pretreatment and posttreatment timepoints. There was no difference in motion between the pretreatment and posttreatment scans (*t* = −0.28, *p* = 0.79).

The CONN toolbox (RRID: SCR 009550) was used to calculate ALFF values within the DLPFC for each participant before and after treatment from the resting state data (Nieto‐Castanon 2020). Values for all voxels within the DLPFC ROI used in the VBM analysis were extracted and averaged, creating a single value at each time point for each participant (Zuo et al. 2010). A paired Wilcoxon Signed Rank Test was performed comparing the pretreatment and posttreatment values. In addition, Spearman's rank correlation coefficients were calculated to examine the relationships between the changes in DLPFC activity and pain outcomes as well as cognitive outcomes, respectively.

### 2.8 Brain Network Community Structure Preprocessing and Analysis

For the community structure analysis, structural imaging preprocessing was completed using SPM 12 and included (1) creating a brain tissue mask through combining white matter, gray matter, and cerebrospinal fluid segmentation; (2) manual quality control (QC) and correction of misclassified brain tissue of T1 brain masks using MRIcron software (https://nitrc.org/projects/mricron); and (3) following QC, masks were warped to MNI standard space (www.mni.mcgill.ca) using Advanced Normalization Tools (ANTs). The functional images also went through standard preprocessing, including (1) removal of first 10 functional scan volumes and realignment to the first volume while correcting for slice‐time differences; (2) reduction of physiological noise by regressing out three mean signals (whole‐brain, white matter, and cerebrospinal fluid) and band‐pass filtering (0.009–0.08 Hz) the images; and (3) performing motion correction to remove scan volumes with extreme motion and global signal distortion (Power et al. 2012). For each functional brain scan, voxel‐wise correlation matrices were generated with each cell representing the Pearson correlation coefficient between the BOLD signal of each voxel pair, making up the voxel‐based functional brain networks. Networks were the thresholded to match the density across subjects with all edges surviving threshold set to 1 and all remaining and negative edges set to 0, resulting in a binary adjacency matrix (Hayasaka and Laurienti 2010).

Using the data obtained from each participant's resting‐state fMRI scan, community structure analyses were performed to divide the network into functional communities. A community is a group of nodes (i.e., a region of the brain) that have high intra‐connectivity within that set of nodes but low connectivity to other nodes in the network. Modularity (Q) is a quantitative measure that can be used to detect the underlying community structure of a network (Newman 2006). The results of these modularity analyses yield maximized modularity, nonoverlapping network community partitions for each individual's brain networks (Newman 2006; Newman and Girvan 2004). For subsequent group‐level analyses, Scaled Inclusivity (SI) was used as a metric to investigate the spatial integrity of the community structure of individual networks across participants (Moussa et al. 2012; Steen et al. 2011). Here, each participant's communities were compared against a priori templates for four functional brain networks, including the default mode network (DMN), central executive network (CEN), salience network (SN), and sensorimotor network (SMN). Outputs from these SI analyses provide a map with the value in each image voxel quantifying the spatial overlap of its community with the a priori network template. A high SI value indicates that an individual's community structure map highly overlaps with the a priori map of a specific network, while a low SI value denotes low overlap between an individual's map and the a priori template.

These analyses were performed using resting‐state data from both before and after treatment interventions.

## 3 Results

### 3.1 Pain‐Related and Cognitive‐Based Outcomes

All six participants enrolled in the study completed the entire testing and intervention protocol. No adverse events were reported. Table 2 displays differences in pain‐related and cognitive outcomes between baseline and post‐intervention. All pain‐related and cognitive outcomes demonstrated improvements following the intervention protocol. However, only changes in PCS, TSK, and SCWT were statistically significant.

**TABLE 2:** Changes in pain and cognitive outcomes.

Outcome measure	Pre m (SD)	Post m (SD)	Percent change (%)	*p* value (pre vs. post)
PCS (0–52)	35.7 (8.2)	15.0 (11.0)	−57.9%	0.028^*^
TSK (16–68)	45.7 (4.9)	33.8 (8.3)	−22.3%	0.028^*^
CSI (0–100)	42.0 (15.2)	37.2 (21.7)	−11.5%	0.249
NPRS (0–10)	3.7 (2.6)	2.0 (1.9)	−45.5%	0.461
PPT (N)	39.9 (17.8)	42.0 (22.8)	+5.5%	0.753
SCWT	291.9 (199.8)	236.9 (146.7)	−18.8%	0.028^*^
CTMT2—Inhibitory	51.8 (5.1)	54.5 (11.4)	+5.1%	0.343
CTMT2—Set Shifting	52.2 (10.67)	55.5 (10.6)	+6.4%	0.093

Abbreviations: CSI, central sensitization inventory; CTMT2—Inhibitory, comprehensive trail making test inhibitory control index; CTMT2—Set Shifting, comprehensive trail making test set shifting index; NPRS, Numeric Pain Rating Scale; PCS, Pain Catastrophizing Scale; PPT, pressure pain threshold; SCWT, Stroop Color and Word Test; TSK, Tampa Scale of Kinesiophobia; WSR, Wilcoxon Signed‐Rank test.

* *p* < 0.05.

### 3.2 VBM Analysis

**FIGURE 1:**
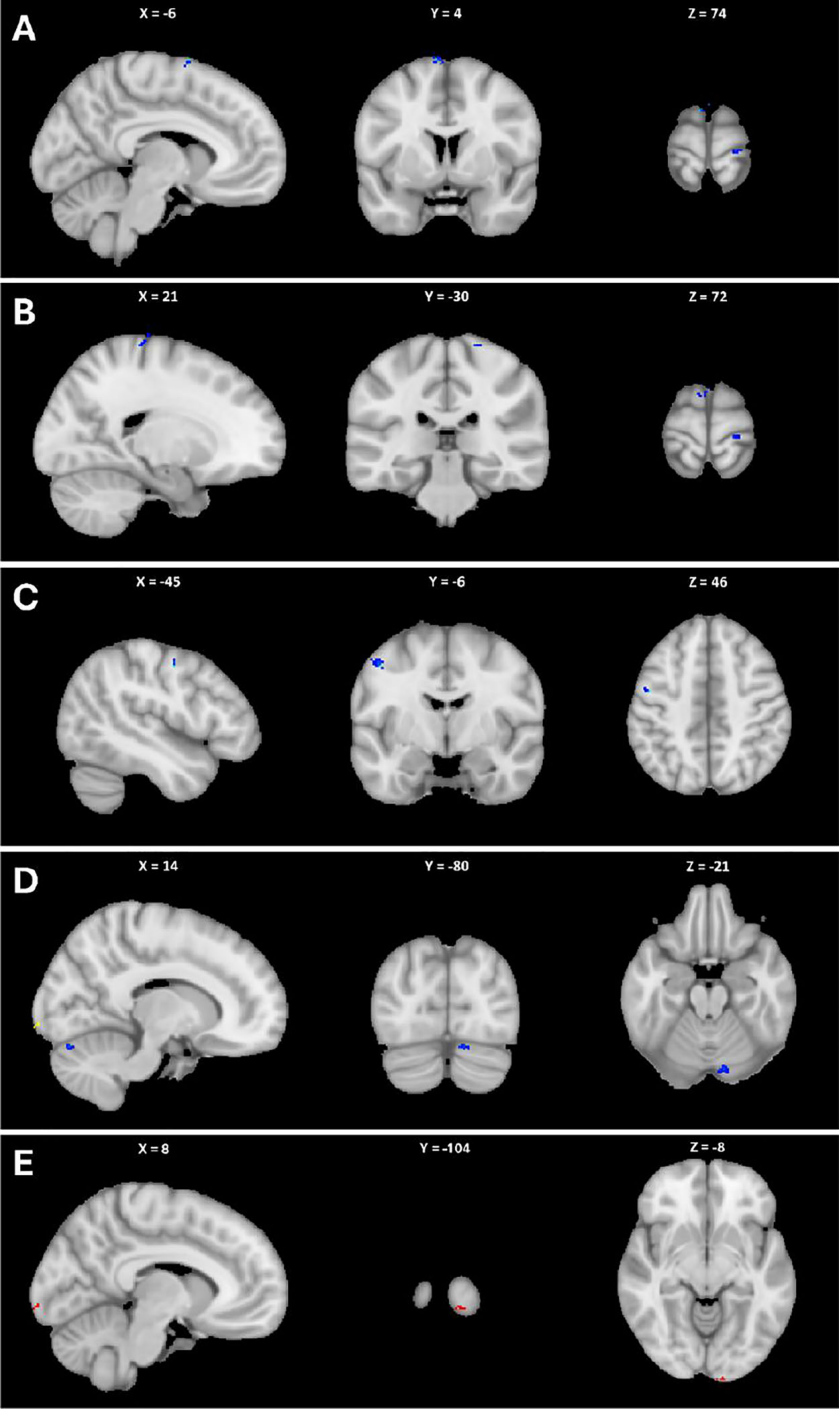
Whole‐brain voxel‐based morphometry results. (A) The left dorsal premotor cortex, (B) the right primary motor cortex, (C) the left primary motor cortex, and (D) the right cerebellum, displayed increased gray matter after treatment relative to before. (E) A single cluster in the occipital cortex, displayed reduced gray matter after treatment relative to before. (clusterwise FWE *p* < 0.05) Results are shown in neurological format.

**FIGURE 2:**
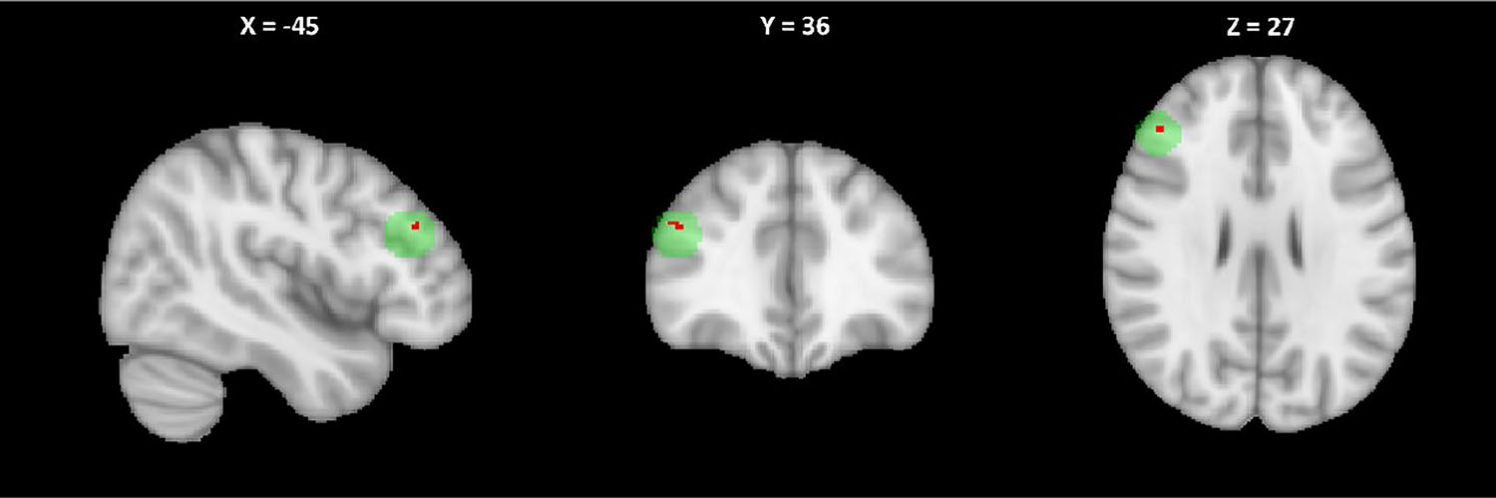
Voxel‐based morphometry identified a 10‐voxel cluster (red) within a 10 mm sphere centered over the left DLPFC at −45, 33, 24 (green), which displayed increased gray matter signal after treatment as compared to before treatment. (*p* < 0.001 uncorrected) Results are shown in native space.

First, an initial whole‐brain analysis was performed. A clusterwise significance threshold of *p* < 0.05 with FWE correction was used. One cluster in the left dorsal premotor cortex (−6, 4 74, Figure 1A) one cluster in the right primary motor cortex (21, −30, 72, Figure 1B), one cluster in the left primary motor cortex (−45, 6, 46, Figure 1C), and one cluster in the right cerebellum (14, −80, −21, Figure 1D) increased in gray matter volume after treatment. A single cluster in the occipital cortex decreased in gray matter volume following treatment (8, −104, −8, Figure 1E). The average gray matter volume change in the largest cluster (left dorsal premotor cortex, peak: −45, −6, 46) identified in the whole brain analysis was calculated for each participant. Additional information regarding these results can be found in Table 3. All participants showed increased signal intensity after treatment relative to before. Using an ROI‐based approach with a spherical implicit mask representing the DLPFC, a 10‐voxel cluster within the left DLPFC displayed greater gray matter after treatment than before treatment, with a peak at −45, 36, 27 (Figure 2). Five of six participants displayed a significant increase in the identified cluster after treatment (Table 4). No voxels had increased gray matter before treatment relative to after treatment at this threshold.

**TABLE 3:** Clusters showing significant changes in gray matter volume between pretreatment and posttreatment with PNE and tDCS (clusterwise FWE < 0.05).

Location	MNI Coordinates of peak voxel			Cluster size	*P‐value (FWE)*
	*X*	*Y*	*Z*		
Left primary motor	−45	−6	46	54	*p* = 0.001
Left dorsal premotor	−6	4	74	37	*p* = 0.001
Right cerebellum	14	−80	−21	31	*p* = 0.039
Right primary motor	21	−30	72	45	*p* = 0.003
Right occipital cortex	8	−104	−8	30	*p* = 0.048

Abbreviations: FWE, family‐wise error rate.

**TABLE 4:** shows changes in gray matter volume in largest cluster identified in the whole brain analysis (left premotor cortex, ‐45, ‐6, 46) and in the a priori left DLPFC analysis. Values are shown as percentage changes averaged across the cluster. Positive percentage change indicates an increase from baseline.

Participant ID	Percent gray matter volume change, left PMC	Percent gray matter volume change, left DLPFC
01	0.04	0.04
02	0.04	0.02
03	0.06	0.34
04	0.01	−0.11
05	0.06	0.19
06	0.06	0.10

### 3.3 Amplitude of Low‐Frequency Fluctuation

**FIGURE 3:**
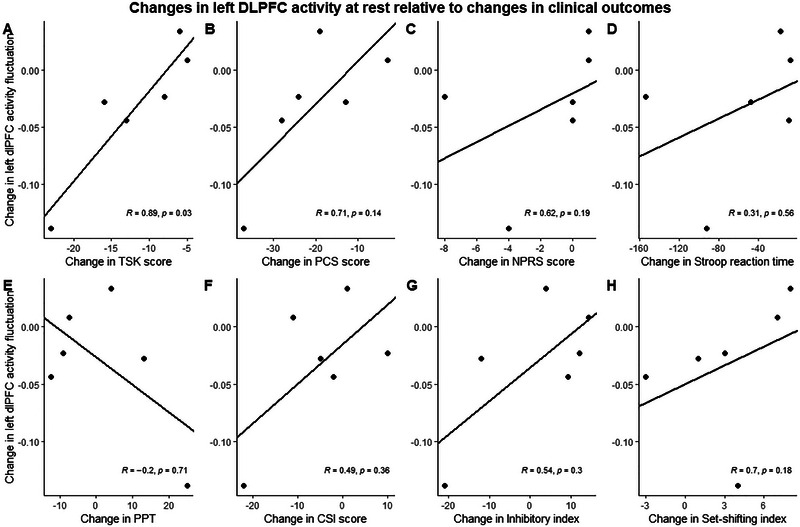
Relationship between changes in left DLPFC activity at rest and clinical measures collected as determined by Spearman's rank correlation coefficient.

There was no difference between pretreatment and posttreatment ALFF (*V *= 16, *p* = 0.31), with four of six participants showing reductions in spontaneous left DLPFC activity after treatment. ALFF changes in the left DLPFC for each participant were plotted against individual changes in clinical outcome measures (Figure 3).

Larger reductions in left DLPFC activity posttreatment were associated with a greater reduction in TSK scores (*R* = 0.89, *p* = 0.03). No other clinical outcome measure changes were significantly related to left DLPFC activity changes; however, the relationship between changes in PCS score (*R* = 0.71, *p* = 0.14) and CTMT set‐shifting index (*R* = 0.7, *p* = 0.18) showed a strong relationship with changes in DLPFC activity fluctuation. Changes in NPRS scores (*R* = 0.62, *p* = 0.19), the Inhibitory Index of the CTMT2 (*R* = 0.54, *p* = 0.3), and CSI scores (*R* = 0.49, *p* = 0.36) showed a moderate relationship with changes in DLPFC activity fluctuation. All other clinical outcomes showed weak or no relationship to changes in DLPFC activity fluctuation from before to after treatment.

### 3.4 Community Structure Analysis

**FIGURE 4:**
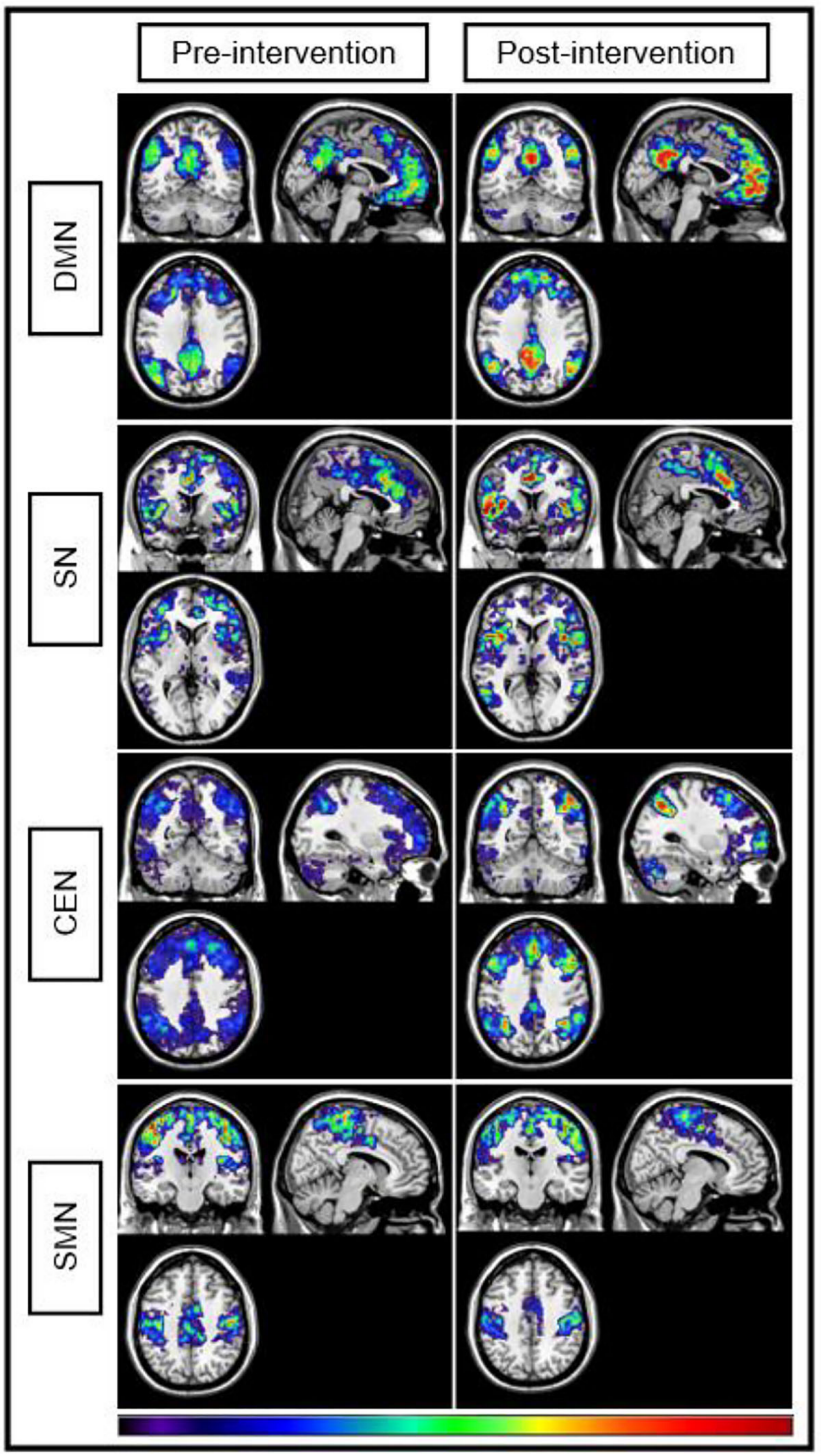
Pre‐ and post‐changes in modularity by network. Warmer colors (red) demonstrate greater consistency within networks while cooler colors (blue) demonstrate lesser consistency. Here, each network shows an increase in modularity (consistency) following the intervention protocol.

Due to the small sample size, there were not sufficient participants to perform a permutation test on the community structure results (Simpson and Laurienti 2015). However, a qualitative comparison of SI maps was performed to identify trends in modularity differences before and after the intervention protocol. Figure 4 displays the averaged SI maps of all six participants before and after the intervention protocol. There were visually apparent increases in community structure integrity following the intervention for the CEN, SN, and DMN. However, community structure integrity within the SMN appears to decrease following the intervention.

## 4 Discussion

To date, studies have combined tDCS to M1 with exercise, visual illusion, or peripheral electrical stimulation for patients with various types of pain (Hazime et al. 2017; Mendonca et al. 2016; Schabrun et al. 2014). Most of these studies have shown greater effects on pain reduction with combined interventions than isolated interventions alone. Three studies have assessed the effects of combining tDCS with CBT for pain. One of these studies investigated a sample of healthy participants, limiting the translation to symptomatic populations (Powers et al. 2018). Luedtke et al. found that tDCS targeting M1 did not influence the outcome of CBT, while others have found tDCS targeting the DLPFC appears to have a priming effect on CBT (Ehsani et al. 2023; Luedtke et al. 2015). While tDCS to M1 has been most often studied, M1 may not be the optimal site when the intervention objective is to modulate pain behaviors. Furthermore, these studies did not use outcome measures that can detect changes in maladaptive pain behaviors.

The results of this exploratory study showed that the combination of tDCS and PNE led to improvement in all pain‐related and cognitive outcomes. Although this study lacked a control group, the results demonstrate the feasibility of the intervention protocol, the positive impact of the intervention, and the lack of treatment‐related side effects. As both pain‐related and cognitive outcomes improved after the intervention, we hypothesize that pain‐related cognitive dysfunction may be a barrier to PNE and that by improving either pain or cognition, the other should improve as well. The results of this study support this idea. Although there are no studies directly assessing PNE's influence on cognitive function, other tDCS literature supports the hypothesis that stimulating a brain region proposed to be involved in recovery associated with PNE (i.e., DLPFC) may allow the individual to be more receptive to this treatment (Ehsani et al. 2023).

Although brain imaging is not a common clinical assessment, research has shown neuroanatomical and neurophysiological alterations associated with the emotional‐affective and cognitive‐evaluative domains of CLBP (De Ridder et al. 2021). Notably, changes occur in these regions equally or more often than in regions directly related to pain processing. Decreased gray matter in DLPFC an area primarily involved in cognitive‐affective pain processing, has been observed in those with CLBP (Apkarian et al. 2004; Hubbard et al. 2014; Seminowicz and Moayedi 2017). In addition, a reduction in gray matter demonstrates a negative correlation between levels of pain catastrophizing and cognitive performance (Apkarian et al. 2004; Ceko et al. 2013; Grachev et al. 2000; Malfliet, Van Oosterwijck, et al. 2017; Seminowicz et al. 2013), whereas an increase in DLPFC activation is correlated with pain reduction (Lorenz et al. 2003; Lorenz et al. 2002). VBM analysis from this study showed an increase in the left DLPFC gray matter volume following the intervention. These changes, along with the aforementioned trends in outcomes, support the findings of Seminowicz et al. (2011), who found a similar response in patients with CLBP 6 months after spine surgery or facet joint injection. This is an exciting finding considering that the success rate of invasive procedures is diminished in a population with high pain behavior levels; thus, the need for better conservative interventions is strong (Attal et al. 2014; Coronado et al. 2015; Giusti et al. 2020). The same author later found that decreasing pain catastrophizing levels was associated with an increase in DLPFC gray matter volume following cognitive behavioral therapy, consistent with our findings (Seminowicz et al. 2013).

How changes in spontaneous DLPFC activity after treatment related to changes in clinical outcomes was also assessed. The correlation between changes in DLFPC activity at rest and TSK scores showed a strong, significant relationship. This means that larger reductions in spontaneous left DLPFC activity after treatment were associated with larger reductions in the TSK over the same time period. These findings are consistent with those of Foss et al., who found higher kinesiophobia was associated with increased DLPFC activation in those with knee pain (Slutsky‐Ganesh et al. 2022). While this highlights a potentially mechanistic relationship, the specific dynamics of this relationship cannot be inferred from this pilot study.

Spatially distributed brain regions interact with others through local and regional networks to perform intricate functions. For instance, the DLPFC is highly interactive within the triple network, a series of networks made up of the (DMN), the (SN), and the (CEN) that support numerous functions, including processing pain experiences (De Ridder et al. 2022; Seeley et al. 2007; Seminowicz and Moayedi 2017). These networks, particularly the DMN and CEN, have been found to undergo neuroplastic changes in the presence of chronic pain (Jones et al. 2020). Similarly, functional changes to these networks also are associated with increased pain catastrophizing and difficulty balancing cognitive demands, yet studies show a reversal of these findings when pain is successfully managed (Čeko et al. 2015; Ellingsen et al. 2021; Hubbard et al. 2014; Lorenz et al. 2003; Lorenz et al. 2002). In this study, consistency of community structure was assessed to describe the relative amount of communication within a network versus other brain regions. Highly modular networks are structured so that communication within a network, or module, is very high, but communication outside (the rest of the brain) of the module is very low. Here, analyses qualitatively demonstrated increased spatial integrity of the modular organization in each network assessed. That is, the nodes in the predefined networks (CEN, DMN, and SN) were more consistently members of the same module following treatment. Conceptually, this represents an increase in connections between nodes within a network, which is indicative of increased functional connectivity and synchrony within these networks. Conversely, we quantitatively identified a decrease in spatial integrity within the SMN after the intervention, indicating decreased modular integrity and lower SMN connectivity and engagement following treatment. It could be hypothesized that this reduction in connectivity may be due to a period of adaptive reorganization as the improvements in the cognitive‐evaluative domains of pain begin to promote alterations in sensorimotor behavior. However, further research is needed to better understand these means.

Alterations in modularity have been found in those with persistent musculoskeletal pain, showing relative increases in inter‐network connections compared to healthy controls in the DMN (Balenzuela et al. 2010; Baliki et al. 2008; Baliki et al. 2014), CEN (Becerra et al. 2014; Tagliazucchi et al. 2010), SN (Larkin et al. 2021), and SMN (Goossens et al. 2018; Pijnenburg et al. 2015). Further, evidence shows increased connections between these networks and regions of the brain involved in the emotional‐affective response to pain, such as the amygdala, cingulate cortex, and insula (De Ridder et al. 2021; De Ridder et al. 2022). This likely results in the elevated sensitivity of the system to pain‐related stimuli with a sustained hypervigilant protective response. The combined tDCS and PNE intervention used in this study appeared to have the ability to reduce the amount of inter‐network communication, thus potentially promoting intra‐network responses. Specifically improving DMN and CEN modularity may reduce the contribution of emotional regions to the self‐referential thought and central executive processes thought to lead to constant, unremitting pain and limited cognitive appraisal (Jiang et al. 2016). By improving network response via tDCS, it was possible that the cognitive functions needed for PNE to have a neuroplastic impact were re‐engaged.

### 4.1 Strengths and Limitations

This exploratory study contains several strengths and limitations. The intervention protocol was designed following evidence‐based guidelines for tDCS and PNE, respectively (Galán‐Martín et al. 2019; Lefaucheur et al. 2017). including consideration of tDCS polarity, intensity, duration, number of intervention sessions, rationale for the treatment target, topics covered in PNE, and duration of PNE sessions. There were no adverse effects to testing or intervention procedures across all participants. The sample size (*n* = 6) limited the outcome assessments to descriptive and nonparametric analysis; thus, the correlations assessed may be unreliable. The lack of a control group also prevented any conclusions from being made regarding the augmentative influence of combining tDCS and PNE versus single intervention alone or the influence of placebo (Colloca 2019, Kong et al. 2007). Medications were not controlled in this study. Many medications taken for the control of pain directly or indirectly influence neuroplasticity and thus may have influenced the effect of treatment. Therefore, we are unable to conclude the influence of the intervention in a controlled fashion. Lastly, because there was no long‐term follow‐up, it is unknown whether treatment effects were sustained.

### 4.2 Clinical Implication

The combination of tDCS and PNE has the potential to become a valuable tool for the management of CLBP. Specifically, the intervention appears to effectively improve emotional‐affective pain behaviors and cognitive function. tDCS is a widely studied modality with evidence supporting clinical benefit for several conditions, such as depression, stroke, aphasia, Alzheimer's, Parkinson's, and schizophrenia (Fregni et al. 2016). PNE has significant support for its use in managing pain behaviors. However, patients with recalcitrant CLBP who are unresponsive to standard interventions and demonstrate centrally mediated barriers to other interventions may benefit from more specific management strategies targeting the central mechanisms involved (i.e., structural and functional brain changes). Future research efforts should include randomized controlled trials to assess the priming influence of tDCS on PNE, include long‐term follow‐ups, and investigate potential dose‐response variables.

## 5 Conclusion

The result of this study showed that five sessions of combined tDCS and PNE intervention improved levels of pain behaviors and cognitive performance as well as demonstrating the ability to promote structural and functional brain changes in those with CLBP. In addition, not only did the intervention protocol used in this study have a potential treatment effect, but it also was a safe intervention because no adverse events were reported. Future RCTs are warranted to investigate the priming influence of tDCS on PNE to determine if there is, in fact, an augmentative effect of the intervention.

## Author Contributions

**Cory Alcon**: conceptualization, methodology, funding acquisition, writing – original draft, investigation, formal analysis, project administration. **Sarah Margerison**: writing – –review and editing, methodology, formal analysis. **Haley Kirse**: investigation, conceptualization, writing – review and editing, methodology, formal analysis. **Christopher Zoch**: investigation. **Paul Laurienti**: conceptualization, investigation, writing – review and editing, formal analysis. **David Seminowicz**: conceptualization, writing – review and editing, formal analysis. **Sharon Wang‐Price**: supervision, investigation, conceptualization, methodology, writing – review and editing, formal analysis.

## Conflicts of Interest

The authors declare no conflicts of interest.

### Peer Review

The peer review history for this article is available at https://publons.com/publon/10.1002/brb3.70543

## Data Availability

The data that support the findings of this study are available on request from the corresponding author. The data are not publicly available due to privacy or ethical restrictions.
